# Aberrant expression of miR‐29b‐3p influences heart development and cardiomyocyte proliferation by targeting NOTCH2

**DOI:** 10.1111/cpr.12764

**Published:** 2020-02-20

**Authors:** Qian Yang, Fang Wu, Yaping Mi, Feng Wang, Ke Cai, Xiaoshan Yang, Ranran Zhang, Lian Liu, Yawen Zhang, Youhua Wang, Xu Wang, Mingqing Xu, Yonghao Gui, Qiang Li

**Affiliations:** ^1^ Translational Medical Center for Development and Disease Institute of Pediatrics Shanghai Key Laboratory of Birth Defect Children's Hospital of Fudan University Shanghai China; ^2^ Cardiovascular Center Children's Hospital of Fudan University Shanghai China; ^3^ Department of Bioscience Bengbu Medical College Bengbu China; ^4^ Longhua Hospital Shanghai University of Traditional Chinese Medicine Shanghai China; ^5^ Cancer Metabolism Laboratory Cancer Institute Fudan University Shanghai China; ^6^ Bio‐X Institutes Key Laboratory for the Genetics of Developmental and Neuropsychiatric Disorders (Ministry of Education) Shanghai Jiao Tong University Shanghai China

**Keywords:** congenital heart disease, microRNA‐29b‐3p, NOTCH2, proliferation

## Abstract

**Objectives:**

microRNA‐29 (miR‐29) family have shown different expression patterns in cardiovascular diseases. Our study aims to explore the effect and mechanism of miR‐29 family on cardiac development.

**Materials and methods:**

A total of 13 patients with congenital heart disease (CHD) and 7 controls were included in our study. Tissues were obtained from the right ventricular outflow tract (RVOT) after surgical resection or autopsy. The next‐generation sequencing was applied to screen the microRNA expression profiles of CHD. Quantitative RT‐PCR and Western blot were employed to measure genes expression. Tg Cmlc2: GFP reporter zebrafish embryos were injected with microRNA (miRNA) to explore its role in cardiac development in vivo. Dual‐luciferase reporter assay was designed to validate the target gene of miRNAs. CCK‐8 and EdU incorporation assays were performed to evaluate cardiomyocyte proliferation.

**Results:**

Our study showed miR‐29b‐3p expression was significantly increased in the RVOT of the CHD patients. Injection of miR‐29b‐3p into zebrafish embryos induced higher mortality and malformation rates, developmental delay, cardiac malformation and dysfunction. miR‐29b‐3p inhibited cardiomyocyte proliferation, and its inhibitor promoted cardiomyocyte proliferation in vitro and in vivo. Furthermore, we identified that miR‐29b‐3p influenced cardiomyocyte proliferation by targeting NOTCH2, which was down‐regulated in the RVOT of the CHD patients.

**Conclusion:**

This study reveals that miR‐29b‐3p functions as a novel regulator of cardiac development and inhibits cardiomyocyte proliferation via NOTCH2, which provides novel insights into the aetiology and potential treatment of CHD.

## 1 INTRODUCTION

Congenital heart disease (CHD) is the most common birth defect with an incidence of 0.41‐10.3 per 1000 live births regarding the severity of CHD, representing a major global health problem.^1^ It is a group of functional and structural malformations of the heart, including valves defects, septa defects, patent foramen ovale, and the heart muscle and outflow tract abnormalities, which cause defects in blood circulation, heart failure and eventual death.^2^ With developments in surgical repair techniques, the number of adult survivors has steadily increased, while the life quality of these survivors decreased.

The heart is the first organ to function during embryogenesis, and its development requires precise temporal and spatial regulation of genetic, environmental and epigenetic factors. The genetic basis of CHD is multifactorial, including chromosomal abnormalities, copy number variants and gene mutations.^3^ Environmental factors contain paternal characteristics and conditions, maternal drug exposures and environmental exposures.^4^ More and more studies have focused on the role of epigenetics on heart development, especially miRNAs. MiRNAs are endogenous small non‐coding RNAs that contain approximately 21‐25 nucleotides and regulate gene expression via imperfect base pairing with 3' UTRs of target mRNAs. Loss of function of enzymes essential for miRNA biogenesis such as Dicer and DGCR8 led to the first insight into the importance of miRNAs in cardiovascular development.^5^, ^6^ Cardiac and skeletal muscle‐specific miR‐1/133 are the most extensively studied miRNAs involved in heart development. Excess miR‐1 caused developmental arrest, thin‐walled ventricles and heart failure through translational inhibition of Hand2, a transcription factor that promoted ventricular cardiomyocyte proliferation and expansion.^7^ MiR‐133a null mice exhibited lethal VSD, dilated cardiomyopathy and heart failure, which can partly be attributed to increased expression of SRF and cyclin D2, the direct targets of miR‐133a.^8^ Cardiac myomiR families (miR‐208a, miR‐208b, miR‐499) participated in the regulation of the host genes switch (Myh6, Myh7 and Myh7b) during development and under stress conditions in a feedback manner.^9^

At the cellular level, CHD has been associated with altered proliferation, migration or differentiation of cardiomyocytes. The proliferation rate of cardiomyocytes is high during early embryogenesis, and it is closely related to cardiac morphology and function. A pool of rapidly proliferating precursor cells outside the heart will give rise to the most of the heart, and foetal heart growth is mainly achieved by cardiomyocyte proliferation.^10^ Both neural‐crest‐ablated and second heart field (SHF)‐ablated embryos, in which the number of cells that contributed to the outflow tract was reduced, resulted in malalignment of the cardiac outflow tract, including coronary artery anomalies, overriding aorta and pulmonary stenosis or atresia.^11^ Reducing cell proliferation by applying the Sonic hedgehog inhibitor cyclopamine also resulted in fewer cells and led to deformities, including pulmonary stenosis, pulmonary atresia and PTA.^12^

MicroRNA‐29 (miR‐29) family regulated cell proliferation, differentiation and apoptosis in cancers, including leukaemia,^13^ liver cancer^14^ and cervical cancer.^15^ Meanwhile, they were essential for extracellular matrix expression during cardiac pathological remodelling.^16^ Several studies have shown different expression patterns of miR‐29 family in CHD patients,^17^, ^18^, ^19^ while whether they participate in the abnormality of heart development and how they function remains unknown. Accordingly, the aim of the present study is to evaluate the role of miR‐29 on heart development and cardiomyocyte proliferation in vitro and in vivo.

## 2 MATERIALS AND METHODS

### 2.1 Patients and controls

Patients with CHD who required surgical reconstruction were from Children's Hospital of Fudan University, Shanghai, China. The diagnosis was based on echocardiography results and confirmed by surgery. Thirteen patients without chromosome abnormalities were included in our study, including 7 (53.8%) males and 6 (46.2%) females, ranging in age from 0.33 to 4.83 years (mean ± SD: 1.8 ± 1.69 years). The control group comprised autopsy specimens from subjects who died as a result of spontaneous abortion at Shanghai General Hospital, Shanghai, China. Specimens from 7 controls, who did not have CHD or a family history of CHD or any other heart disease, included 3 (42.9%) males and 4 (57.1%) females, ranging in age from 23 to 27 weeks (mean ± SD: 25.29 ± 1.70 weeks). Characteristics of the study subjects are summarized in Table S1. All cardiac tissues were obtained from the RVOT immediately after surgical resection or autopsy and stored in liquid nitrogen for RNA/DNA/protein extraction or in formalin for immunohistochemical analysis. This study was approved by the local ethics committee of Children's Hospital of Fudan University and Shanghai General Hospital. Written informed consents were obtained from the parents of all study subjects.

### 2.2 RNA extraction and quantitative RT‐PCR

Total RNA was extracted with the miRNeasy Mini Kit (Qiagen) following the manufacturer instructions. About 500 ng RNA was reverse transcribed via the PrimeScript RT reagent kit (Takara), and the amplification was performed with SYBR premix Ex Taq (Takara). Primer sequences are listed in Table S2. miRNAs were reverse transcribed by the miRcute plus miRNA first‐strand cDNA synthesis kit (Tiangen) and quantified by the miRcute Plus miRNA qPCR detection kit (Tiangen) with specific primers (Tiangen) using a LightCycler 480 detection system. The primer for miR‐29b‐3p in human, rat, mouse and zebrafish is 5′‐GAGGTAGCACCATTTGAAATCAGTGT‐3′. The primer for hsa‐miR‐29b‐1‐5p, dre‐miR‐29b3‐5p and mmu‐miR‐29b‐1‐5p is 5′‐GGAGCTGGTTTCATATGGTGGTTT‐3′. The primer for rno‐miR‐29b‐1‐5p is 5′‐AGGGTTGGGGTGGTTTAGATTT‐3′. The relative mRNA and miRNA quantifications were normalized against β‐actin or U6.

### 2.3 miRNA sequencing

Small RNA libraries were constructed using the Small RNA Sample Preparation kit (Illumina). A 5′ adapter and a 3′ adapter were ligated to the small RNA. Reverse transcription was used to obtain sufficient amount of template for sequencing. The final amplification products were gel purified and sequenced on the Illumina HiSeq TM2500/MiSeq.

### 2.4 Cell culture and transfection

The mouse atrial cardiomyocyte‐derived cells HL1 were kindly provided by Prof. Duan Ma, Fudan University. The embryonic rat ventricular cardiomyocyte‐derived cells H9c2 and HEK 293 cells were purchased from the Cell Bank of the Chinese Academy of Sciences, Shanghai, China. All cells were cultured in Dulbecco's modified Eagle's medium which contained 10% foetal bovine serum and 1% penicillin‐streptomycin at 37°C under humidified conditions of 95% air and 5% CO_2_. The sequence of miR‐29b‐3p was conserved in human, rat, mouse and zebrafish (Table S3). miRNA mimics and inhibitors were purchased from GenePharma, and their sequences are listed in Table S4. NOTCH2 siRNA was purchased from GenePharma, and the sequences were 5′‐GCAUCUGUCCCUUGGGCUATT‐3′ and 3′‐UAGCCCAAGGGACAGAUGCTT‐5′. Mouse NICD2 (Notch2 intracellular domain) gene was amplified by PCR from the genomic DNA of HL1 cells and subcloned into pCHD‐puro retroviral vector. When cells grew to 50%‐70% confluence, miRNA mimic or inhibitor was transfected by Lipofectamine™ RNAiMax and plasmids were transfected by Lipofectamine™ 3000 (Life Technologies) according to the manufacturer's instructions.

The medium was changed 24 hours later and cells were harvested 48 hours later.

### 2.5 Immunohistochemistry

Immunohistochemical analysis was performed on tissue serial sections of formalin‐fixed paraffin‐embedded tissue blocks from patients and controls as described previously. After dewaxing, rehydrating and heat‐induced antigen retrieval, endogenous peroxidase activity and non‐specific binding were blocked with 3% H_2_O_2_ and non‐immunogenic sera, respectively. Sections were incubated with 5% BSA for 1 hour at room temperature and primary antibody targeting NOTCH2 overnight at 4°C (1:1000 dilution; Cell Signaling Technology). The slides were then stained using a VECTASTAIN ABC kit (Vector Laboratories) and a DAB Peroxidase Substrate kit (Vector Laboratories) according to the manufacturers' instructions.

### 2.6 Western blot analysis

Total protein was obtained by RIPA lysis buffer (Beyotime) with protease inhibitor cocktail (Thermo Fisher), and protein concentrations were measured by Pierce BCA Protein Assay Kit (Thermo Fisher). A total of 30‐40 ng protein extracts were loaded onto SDS‐PAGE gels (6%‐12%) and then transferred to PVDF membranes. PVDF membranes were blocked by 5% w/v non‐fat dry milk which was dissolved in tris‐buffered saline solution containing 0.1% tween‐20 (TBS‐T) for 2 hours. The PVDF membranes were then incubated with the primary antibodies at 4°C overnight: anti‐NOTCH2 (1:4000 dilution; Cell Signaling Technology), anti‐phospho‐Histone H3 (PHH3) (1:2000 dilution; Cell Signaling Technology), anti‐PCNA (1:2000 dilution; Cell Signaling Technology) and anti‐β‐actin (1:4000 dilution; Cell Signaling Technology). After three washing with TBS‐T, the PVDF membranes were incubated with HRP‐labelled goat anti‐rabbit IgG secondary antibody (1:8000 dilution; Abcam) for 1 hour at room temperature. After the final washing with TBS‐T, protein bands were visualized with ECL (Merck Millipore) reagents.

### 2.7 Luciferase assay

Bioinformatic tools (PicTar and TargetScan algorithms) were used to predict the targets of miR‐29b‐3p. The NOTCH2 3′ UTR from human/mouse/rat genomic DNA was cloned into XhoI and NotI sites downstream of Renilla luciferase in the psiCHECK‐2 vector (Promega), while the firefly luciferase gene was used as an internal control. Mutation of the NOTCH2 3′ UTR was performed using Fast Mutagenesis System (TransGen Biotech). The PCR primer is listed in Table S5. For analysis of luciferase activity, HEK293, HL1 and H9c2 cells were cultured in 96‐well plates and co‐transfected with 100 ng of psiCHECK‐2 vector containing the 3′ UTR of NOTCH2 (WT or MUT) and 20 pmol of miRNA mimic per well. The four groups were psiCHECK‐2‐NOTCH2‐WT+ miR‐NC mimic, psiCHECK‐2‐NOTCH2‐WT+ miR‐29b‐3p mimic, psiCHECK‐2‐NOTCH2‐MUT+ miR‐NC mimic and psiCHECK‐2‐NOTCH2‐MUT+ miR‐29b‐3p mimic. The luciferase analysis was performed 24 hours later with the Dual‐Luciferase Reporter Assay (Promega). After lysed for 15 minutes at room temperature, the relative luciferase activity was obtained after normalization to firefly activity.

### 2.8 Cell viability assay

Cell viability was measured by cell counting kit‐8 (Dojindo Laboratories). A total of 4 × 10^3^ cells were seeded into a 96‐well plate per well, and 10 µL of CCK‐8 solution was added into 100 µL of culture medium. Cells were incubated at 37°C for 2.5 hours, and the OD was measured at 450 nm.

### 2.9 EdU incorporation assay

The EdU incorporation assay was carried out according to the manufacturer's instructions (Life Technologies). Cardiomyocytes were fixed with 4% paraformaldehyde for 15 minutes, permeated in 1.0% Triton X‐100 for 20 minutes and then incubated with 10 μmol/L EdU for 1.5 hours. Following three washing with PBS, the click‐it reaction cocktail was added into culture medium and then incubated for 30 minutes at room temperature. Cardiomyocytes were stained with Hoechst 33342 for 30 minutes after three washing with PBS and visualized under a fluorescence microscope (Olympus Corporation). The cell proliferative rate was calculated as the proportion of nucleated cells that incorporated EdU in 10 high‐power fields per well.

### 2.10 Zebrafish embryology and miRNA injection

Zebrafish husbandry, embryo collection and maintenance were carried out according to accepted standard operating procedures. The embryos of cardiac myosin light chain 2: EGFP transgenic line [Tg (cmlc2: EGFP)] were maintained at 28.5°C with a 14:10‐h light:dark cycle. About 5 µmol/L*3 nL miRNA was injected into the yolk of zebrafish embryos at the 1‐4 cell stage. Embryos were visualized with the Leica M205 FA digital camera.

### 2.11 General morphology score system

The general morphology score (GMS) system was applied to assess the development of zebrafish, which included tail detachment, somite formation, eye development, movement and heart beat, etc.^20^

### 2.12 Heart rate measurement

The times of heart beats in 15 seconds were counted from a video, and the heart rate was multiplied by four.

### 2.13 Quantifying shortening fraction

The maximum ventricular systole and ventricular diastole was measured by ImageJ. The shortening fraction (%) of the ventricle was calculated as follows: 100 × (width at diastole − width at systole)/(width at diastole)%.

### 2.14 Immunofluorescence and confocal microscopy

Tg (cmlc2: EGFP) embryos were fixed in 4% paraformaldehyde at 4°C overnight. Fixed embryos were exposed to collagenase II for 2 hours at room temperature and then stained with anti‐PCNA (1:100 dilution; Cell Signaling Technology) overnight at 4°C. After being washed with TBS‐T for 1 hour, the embryos were stained with Alexa Fluor 546 goat anti‐rabbit IgG (Invitrogen) overnight at 4°C. After being washed with TBS‐T for 1 hour, the embryos were incubated with DAPI (1:200; Beyotime) for 2 hours at room temperature. Embryonic hearts were then imaged using confocal microscopy (Leica TCS SP8). The total number of proliferating cardiomyocytes per heart was determined and summed from each image of Z‐disc sections for each group.

### 2.15 Statistics

All data were presented as the mean ± SEM. Associations between miR‐29b‐3p expression and the severity of CHD were assessed by Spearman rank correlations. The scores aimed to quantify the development of zebrafish embryos at different endpoints were assessed by Wilcoxon signed rank sum test. Differences in the expression of genes and proliferation rate were analysed with Student's *t* test. The data were analysed using GraphPad Prism software (version 5.00; GraphPad software, Inc).

## 3 RESULTS

### 3.1 The expression of miR‐29b‐3p was higher in the RVOT of CHD patients

**Figure 1:**
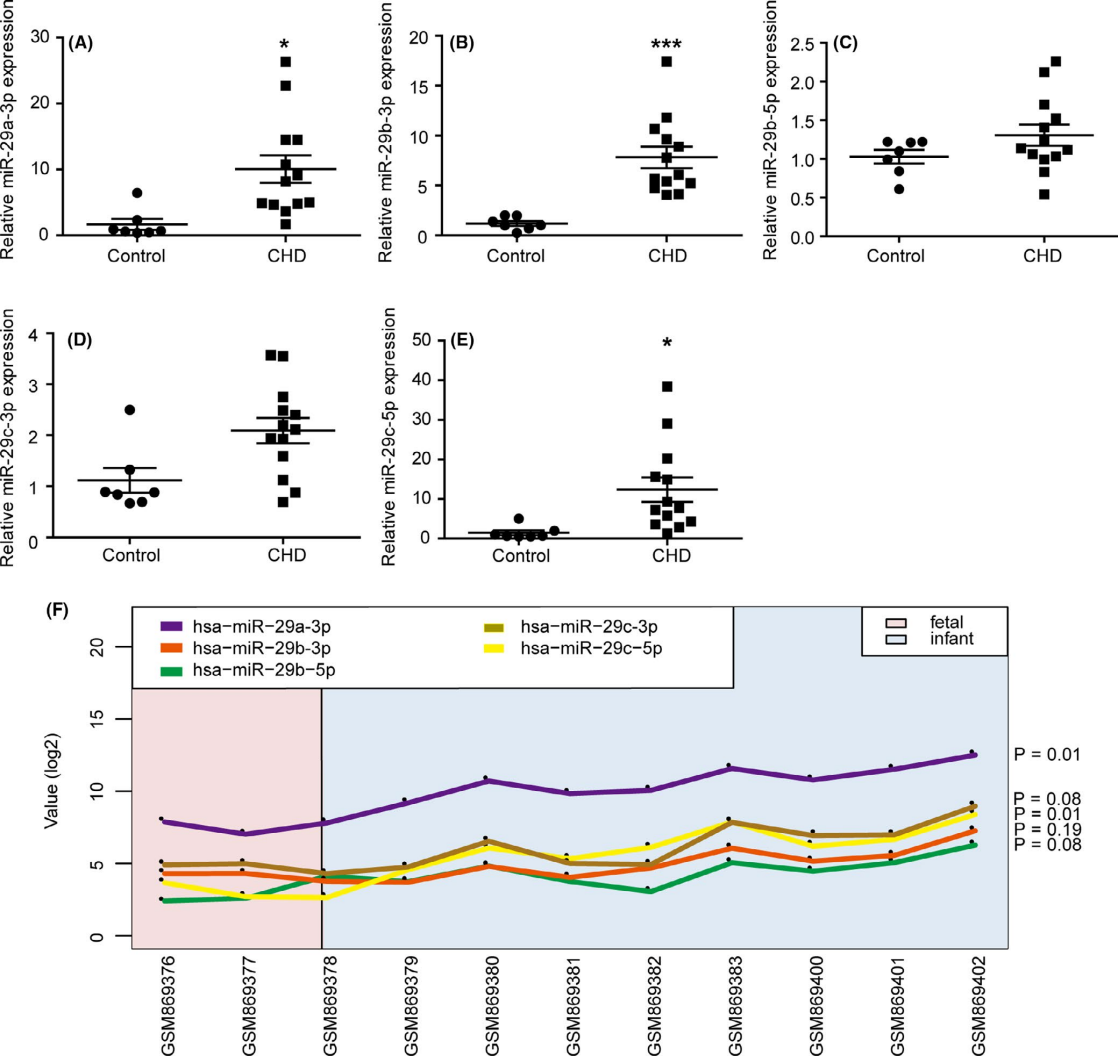
Relative expression of miR‐29b‐3p was higher in the right ventricular outflow tract (RVOT) of congenital heart disease (CHD) patients. The expression of miR‐29a‐3p (A), miR‐29b‐3p (B), miR‐29b‐5p (C) miR‐29c‐3p (D), miR‐29c‐5p (E) in the RVOT of CHD patients (n = 13) and controls (n = 7) was determined via miRNA stem loop RT‐PCR. MiRNAs were normalized to U6 expression. The expressions of miR‐29a‐3p, miR‐29b‐3p and miR‐29c‐5p were up‐regulated in the RVOT of the CHD patients than the controls. F, The influence of age to the expression of miR‐29 family. Only miR‐29b‐3p expression was not altered as age increased among the 3 up‐regulated miR‐29 family members. The data are presented as the mean ± SEM. Statistical significance is shown as *P < .05 vs controls, **P < .01 vs controls, and ***P < .001 vs controls

To detect the dysregulated miRNAs in the RVOT of the CHD group and the control group, we performed miRNA sequencing to evaluate the miRNA profiles of heart tissues from 3 patients and 3 controls. According to the results of miRNA sequencing, we found that miR‐29 family members were the most significantly up‐regulated miRNAs, including miR‐29a‐3p, miR‐29b‐3p, miR‐29c‐5p and miR‐29c‐3p (Table 1). We validated the expression of miR‐29 family members in the RVOT of 13 patients and 7 controls by qRT‐PCR. The results showed that the expression of miR‐29a‐3p, miR‐29b‐3p, miR‐29c‐5p was significantly higher in the case group, while the expression of miR‐29b‐5p, miR‐29c‐3p was not different (Figure 1A‐E). As there was an age difference between the case group and the control group and it was difficult to obtain age‐matched healthy controls, we analysed the data in GEO database to compare the expression of miR‐29 family at different developmental stages in human heart tissues (GSM869376‐GSM869383, GSM869400‐GSM869402). The 3 foetal hearts (about 90‐day gestation) were from the Central Laboratory for Human Embryology at the University of Washington. The 8 normally developing infant hearts (3 males, 5 females) were obtained from LifeNet Health (http://www.lifenethealthy.org). There were no heart malformations in all samples. The results showed that the expression of miR‐29a‐3p (*P* = .01) and miR‐29c‐5p (*P* = .01) changed as the age increased, while the expression of miR‐29b‐3p was not altered (*P* = .19) (Figure 1F). Thus, we concluded that miR‐29b‐3p expression was higher in the RVOT of the case group.

**Table 1:** miRNAs with significant up‐regulation in congenital heart disease

miRNA	Fold‐change	*P*‐value
hsa‐miR‐29a‐3p	4.5525	6.95E‐66
hsa‐miR‐23b‐3p	2.4587	1.60E‐50
hsa‐miR‐221‐5p	3.5181	1.73E‐50
hsa‐miR‐29b‐3p	4.7809	8.60E‐43
hsa‐miR‐27a‐3p	2.4816	1.26E‐41
hsa‐miR‐302d‐3p	2.4812	2.42E‐40
hsa‐miR‐302b‐3p	2.6286	2.34E‐30
hsa‐miR‐95‐3p	2.287	6.96E‐28
hsa‐miR‐3074‐3p	2.6645	1.42E‐26
hsa‐miR‐29c‐5p	4.2146	5.72E‐26
hsa‐miR‐499a‐5p	1.5899	3.16E‐25
hsa‐miR‐499b‐3p	1.6007	9.38E‐25
hsa‐miR‐29c‐3p	4.3671	1.67E‐24
hsa‐miR‐24‐3p	2.101	1.22E‐23
hsa‐miR‐664a‐3p	2.0543	1.22E‐23
hsa‐miR‐24‐2‐5p	1.6882	1.96E‐23
hsa‐miR‐3074‐5p	2.0994	7.86E‐23
hsa‐miR‐302a‐3p	3.0517	1.11E‐22
hsa‐miR‐222‐3p	3.2499	4.51E‐22
hsa‐miR‐302c‐3p	3.4016	2.08E‐21

We detected the ROC curve for diagnostic value of miR‐29b‐3p expression level for CHD. As shown in Figure S1, the area under the ROC curve was 0.92 (95% CI = 0.79‐1). This result showed that the expression of miR‐29b‐3p was helpful for the diagnosis of CHD. However, due to the limited sample size, the results should be verified by studies with enlarged sample size. We compared the echocardiography parameters with the miR‐29b‐3p expression level in the case group. None of these parameters exhibited a significant correlation with miR‐29b‐3p expression, which meant its expression may not be correlated with the severity of CHD (Figure S2).

### 3.2 miR‐29b‐3p induced cardiac malformation in zebrafish embryos

**Figure 2:**
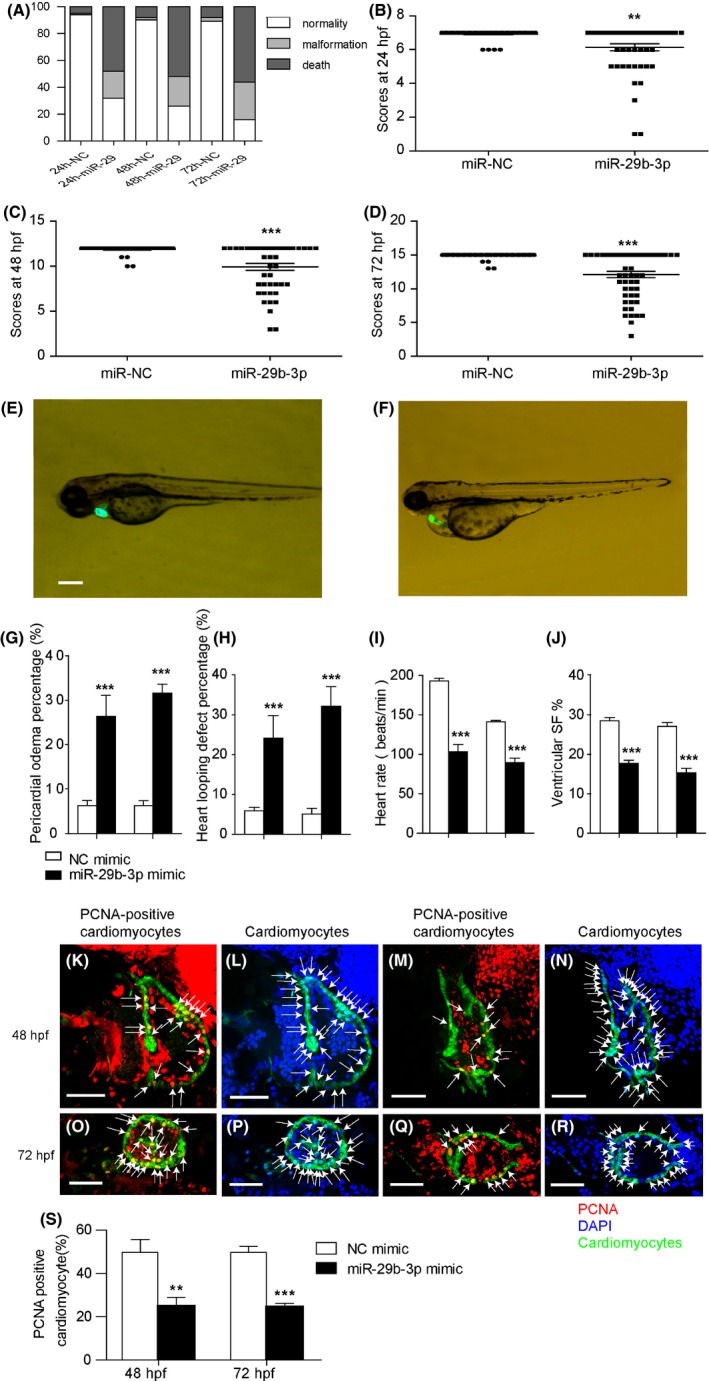
MiR‐29b‐3p injection disturbed the development of zebrafish heart. A, MiR‐29b‐3p injection led to a higher mortality and malformation rate in zebrafish embryos from 24 to 72 hpf (n > 100). B‐D, The general morphology score system was used to evaluate the development of zebrafish embryos. MiR‐29b‐3p injection induced a developmental delay in zebrafish embryos at 24, 48 and 72 hpf (n > 50). E, Normal heart morphology of 72 hpf zebrafish embryos injected with miR‐NC mimic. F, Abnormal heart morphology of 72 hpf zebrafish embryos injected with miR‐29b‐3p mimic, including pericardial oedema and looping defects. Cardiac visualization under bright‐field microscopy, with the photographs showing heart morphology (bar = 100 µm). G and H, MiR‐29b‐3p injection caused a higher degree of pericardial oedema and looping defects in zebrafish embryos (n > 50). I and J, MiR‐29b‐3p injection induced a decrease in heart rate and ventricular shortening fraction (n > 50). K‐N, PCNA in the hearts of zebrafish embryos injected with NC mimic (K, L) or miR‐29b‐3p mimic (M, N) at 48 hpf. O‐R, PCNA in the hearts of zebrafish embryos injected with NC mimic (O, P) or miR‐29b‐3p mimic (Q, R) at 72 hpf. Arrows indicated PCNA‐positive cardiomyocytes (K, M, O, Q) and the nuclei of cardiomyocytes (L, N, P, R). The proliferation index was calculated by dividing the number of PCNA‐positive cardiomyocytes by the total number of cardiomyocytes at the largest anatomical level of the ventricle. Cmlc2‐positive (green) staining indicated cardiomyocytes, nuclei were labelled with DAPI (blue), and PCNA‐positive (red) staining indicated proliferative cells (bar = 50 µm). S, Quantitative assessment of the proliferation index of PCNA‐positive cardiomyocytes (n = 5‐7). The data are presented as the mean ± SEM. Statistical significance is shown as *P < .05 vs controls, **P < .01 vs controls, and ***P < .001 vs controls

To evaluate the role of miR‐29b‐3p during cardiac development, we injected miR‐29b‐3p mimic or miR‐NC mimic into zebrafish embryos. The expression of miR‐29b‐3p and miR‐29b‐5p in zebrafish embryos was detected by qRT‐PCR. The results showed that miR‐29b‐3p was significantly up‐regulated in zebrafish embryos injected with miR‐29b‐3p mimic at 24, 48 and 72 hpf (Figure S3A), while the expression of miR‐29b‐5p was not altered (Figure S3B). The mortality and malformation rates of the miR‐29b‐3p group were increased markedly compared with the control group (Figure 2A). We adopted the GMS system to assess the effects of miR‐29b‐3p on zebrafish development. Embryos injected with miR‐29b‐3p mimic showed developmental delay when compared with the miR‐NC group at different time points (Figure 2B‐D). Moreover, miR‐29b‐3p caused multiple malformations including body malformation and cardiac deformations in zebrafish embryos. Cardiac deformations included pericardial oedema, looping abnormality, bradycardia and decreased ventricular shortening fraction. Figure 2E presented the normally developing zebrafish embryo at 72 hpf in the control group, and Figure 2F presented the malformed zebrafish embryo with pericardial oedema and looping abnormality in the miR‐29b‐3p group. MiR‐29b‐3p treatment led to a higher percentage of embryos with pericardial oedema and looping abnormality (Figure 2G,H). Meanwhile, it decreased the heart rate and ventricular shortening fraction of zebrafish embryos (Figure 2I,J). Video recordings of cardiac contractions of the zebrafish embryos are presented in Videos S1 and S2. Taken together, these results demonstrated that miR‐29b‐3p injection impaired zebrafish cardiac morphology and function.

### 3.3 MiR‐29b‐3p inhibited cardiomyocyte proliferation in vivo and in vitro

To explore the effects of miR‐29b‐3p on cardiomyocyte proliferation in vivo, we injected miR‐29b‐3p mimic or miR‐NC mimic into zebrafish embryos to evaluate cardiac proliferation. Proliferative cardiomyocytes were identified by anti‐PCNA staining. PCNA was a DNA replication marker. Zebrafish injected with miR‐29b‐3p mimic showed a significant decrease in the number of proliferative cardiomyocytes at 48 and 72 hpf (Figure 2K‐S). These results demonstrated that miR‐29b‐3p inhibited cardiomyocyte proliferation in vivo.

**Figure 3:**
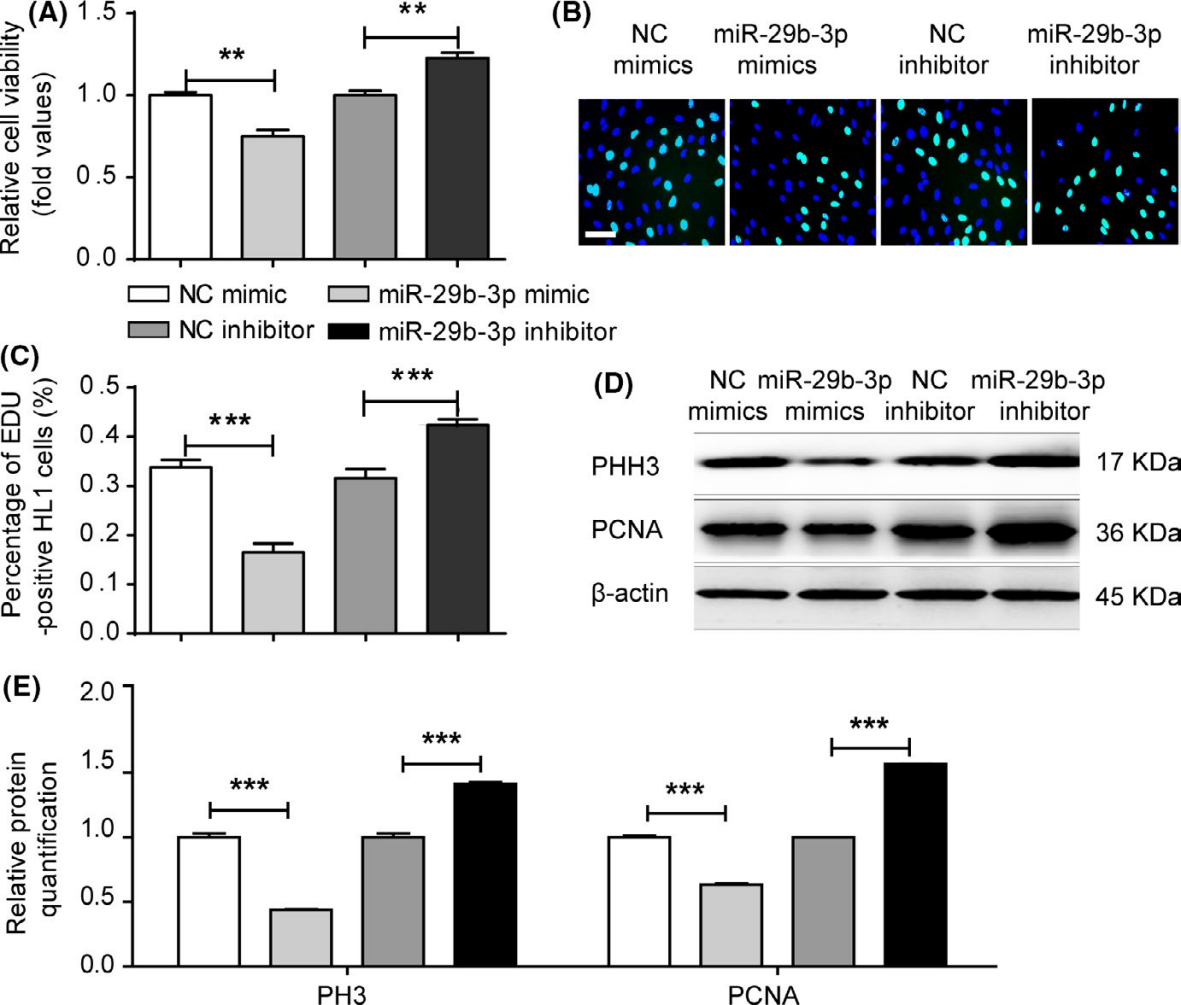
MiR‐29b‐3p decreased HL1 cell proliferation. A, Cell viability assay was assessed via CCK‐8 assays. Cell viability was lower in the miR‐29b‐3p mimic group and higher in its inhibitor group. B and C, EdU incorporation assay of cardiomyocytes. Microscopy images illustrated the EdU and Hoechst staining of cardiomyocytes after transfection with miR‐29b‐3p mimic or inhibitor (bar = 50 μm). The percentage of proliferating cardiomyocytes was calculated (n > 1000). EdU incorporation was decreased in the miR‐29b‐3p mimic group and enhanced in its inhibitor group. D, WB analysis of PHH3 and PCNA. E, Relative quantification of PHH3 and PCNA protein. The expressions of PHH3 and PCNA protein were down‐regulated in the miR‐29b‐3p mimic group and up‐regulated in its inhibitor group. The data are presented as the mean ± SEM. Statistical significance is shown as *P < .05 vs controls, **P < .01 vs controls, and ***P < .001 vs controls

To explore the role of miR‐29b‐3p in cardiomyocyte proliferation in vitro, we transfected HL1 cells with miR‐29b‐3p mimic or inhibitor and measured the expression of miR‐29b‐3p and miR‐29b‐5p by qRT‐PCR. The results showed that the expression of miR‐29b‐3p was significantly increased in the mimic group and decreased in the inhibitor group, while the expression of miR‐29b‐5p was not influenced by miR‐29b‐3p mimic or inhibitor (Figure S4). CCK‐8 (Figure 3A) and EdU (Figure 3B,C) assays showed that miR‐29b‐3p mimic decreased while its inhibitor significantly promoted cell proliferation. Furthermore, we analysed the expression of PCNA and PHH3, which were down‐regulated in the miR‐29b‐3p mimic group and up‐regulated in the miR‐29b‐3p inhibitor group (Figure 3D,E). These results indicated that miR‐29b‐3p inhibited cardiomyocyte proliferation, while its inhibitor promoted proliferation in vitro.

### 3.4 The expression of NOTCH2 was decreased in the RVOT of CHD patients

**Figure 4:**
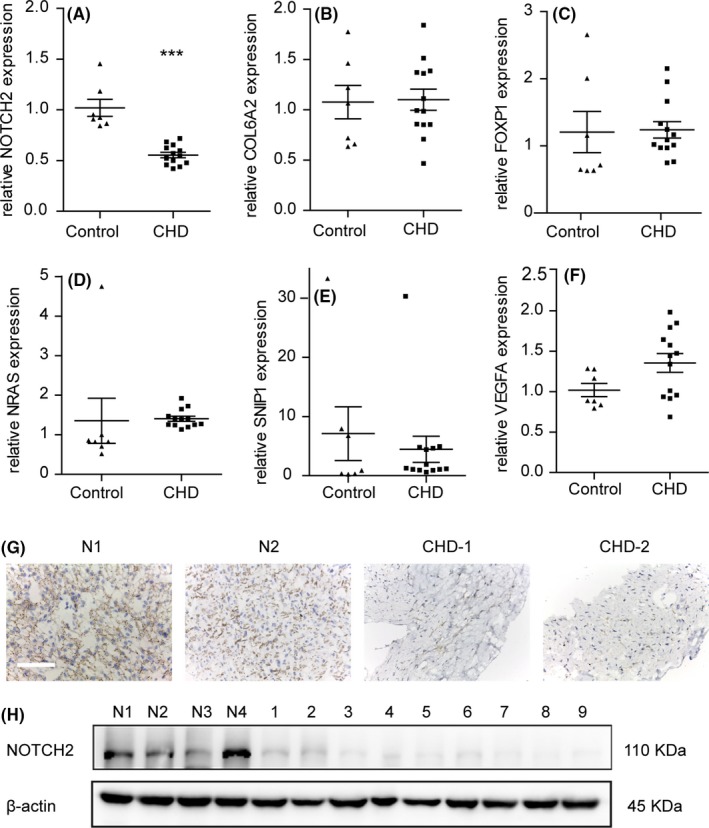
The expression of NOTCH2 was lower in the right ventricular outflow tract of congenital heart disease (CHD) patients. A‐F, Relative mRNA expressions of NOTCH2, COL6A2, FOXP1, NRAS, SNIP1 and VEGFA were analysed using real time RT‐PCR and normalized to β‐actin. NOTCH2 exhibited lower mRNA expression in the CHD patients (n = 13) than in the controls (n = 7). G, Immunohistochemical analysis of NOTCH2 in the RVOT of the controls (N1, N2) and CHD patients (CHD‐1, CHD‐2). The heart tissues in the CHD patients showed weak cytoplasmic staining for NOTCH2 (bar = 100 µm). H, NOTCH2 protein was assessed in the CHD patients (n = 9) and the controls (n = 4) with WB. NOTCH2 protein expression was lower in the CHD patients than in the controls. The data are presented as the mean ± SEM. Statistical significance is shown as *P < .05 vs controls, **P < .01 vs controls, and ***P < .001 vs controls

To explore the biological mechanism of miR‐29b‐3p in cardiomyocyte proliferation, we used bioinformatic tools to predict the targets of miR‐29b‐3p. Since CHD was a manifestation of abnormal heart development, we focused on genes associated with heart development and disease. The mRNA expressions of the predicted targets (NOTCH2, COL6A2, FOXP1, NRAS, SNIP1 and VEGFA) were assessed in the RVOT of the CHD group and the control group. The results showed that among the six targets, only NOTCH2 exhibited significantly lower mRNA expression levels in CHD patients (Figure 4A‐F). To validate the expression of NOTCH2 at protein level, immunohistochemistry and Western blotting were performed. Similar to the qRT‐PCR results, NOTCH2 protein expression was also decreased in CHD patients (Figure 4G,H).

In order to exclude the impact of age difference on the expression of NOTCH2, we analysed the expression of NOTCH2 at different developmental stages in mouse heart tissues. We collected the RNA sequencing data from GEO database and compared the expression of NOTCH2 at E12.5, E14.5, E18.5 and 3 days after birth (GSM1246323‐GSM1246325, GSM1246329‐GSM1246331, GSM1246335‐GSM1246337 and GSM1246341‐GSM1246343). The results showed that the expression of NOTCH2 did not alter as age increased (*P* = .18) (Figure S5).

### 3.5 NOTCH2 was a direct target of miR‐29b‐3p

**Figure 5:**
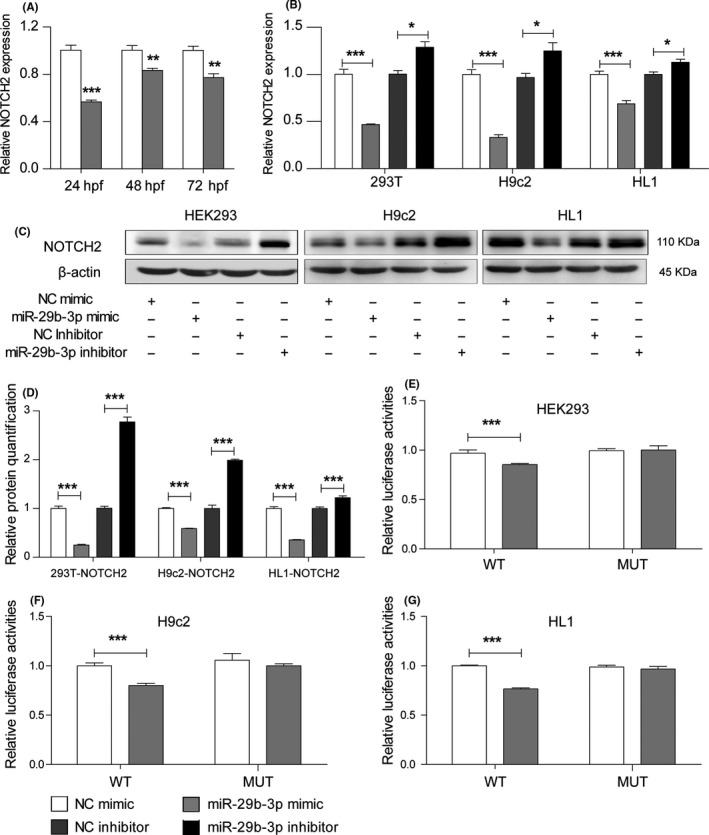
MiR‐29b‐3p regulated NOTCH2 by directly targeting its 3' UTR. A, The mRNA expression of NOTCH2 was decreased in zebrafish embryos injected with miR‐29b‐3p mimic vs NC mimic from 24 to 72 hpf. B, qRT‐PCR results showed that NOTCH2 mRNA expression was decreased in the miR‐29b‐3p mimic group and increased in the inhibitor group. C, WB results showed that NOTCH2 protein expression was lower in cells transfected with miR‐29b‐3p mimic and higher in cells transfected with miR‐29b‐3p inhibitor. D, Relative quantification of NOTCH2 protein. E, HEK‐293 cells was co‐transfected with psiCHECK‐2‐NOTCH2‐3'UTR‐WT‐Human (100 ng) or psiCHECK‐2‐NOTCH2‐3'UTR‐MUT‐Human (100 ng) and miR‐29b‐3p mimic (20 pmol) or NC mimic (20 pmol). F, H9c2 cells were co‐transfected with psiCHECK‐2‐NOTCH2‐3'UTR‐WT‐Rat (100 ng) or psiCHECK‐2‐NOTCH2‐3'UTR‐MUT‐Rat (100 ng) and miR‐29b‐3p mimic (20 pmol) or NC mimic (20 pmol). G, HL1 cells were co‐transfected with psiCHECK‐2‐NOTCH2‐3'UTR‐WT‐Mouse (100 ng) or psiCHECK‐2‐NOTCH2‐3'UTR‐MUT‐Mouse (100 ng) and miR‐29b‐3p mimic (20 pmol) or NC (20 pmol) mimic. MiR‐29b‐3p decreased the relative luciferase activity in the WT group but had no effect in the MUT group. The data are presented as the mean ± SEM. Statistical significance is shown as *P < .05 vs controls, **P < .01 vs controls, and ***P < .001 vs controls

To explore the regulatory effect of miR‐29b‐3p on NOTCH2 expression, we introduced miR‐29b‐3p mimic or inhibitor into zebrafish embryos and cell lines. mRNA expression of NOTCH2 in zebrafish embryos injected with miR‐29b‐3p mimic was down‐regulated at 24, 48, 72 hpf (Figure 5A). Meanwhile, its expression in HEK293, H9c2 and HL1 cells was decreased in the miR‐29b‐3p mimic group and increased in its inhibitor group (Figure 5B). Moreover, the WB results showed that NOTCH2 protein level was significantly altered by miR‐29b‐3p mimic or inhibitor in all cell lines (Figure 5C,D). To determine whether NOTCH2 was a direct target of miR‐29b‐3p, we assessed the interaction between miR‐29b‐3p and the 3' UTR of NOTCH2. MiR‐29b‐3p was broadly conserved, and the “seed sequence” was predicted to bind with the 3' UTR of NOTCH2 among different species (Figure S6). HEK293, HL1 and H9c2 were co‐transfected with miRNA mimic and the luciferase reporter vector for the 3' UTR of NOTCH2 for each species. MiR‐29b‐3p mimic inhibited the relative luciferase activity of the NOTCH2 3' UTR‐WT vector, and the inhibitory effect of the miR‐29b‐3p mimic was abrogated after co‐transfection with the NOTCH2 3′ UTR‐MUT vector (Figure 5E‐G). These results indicated that NOTCH2 was a specific and direct target of miR‐29b‐3p.

### 3.6 MiR‐29b‐3p inhibited cardiomyocyte proliferation by targeting NOTCH2

**Figure 6:**
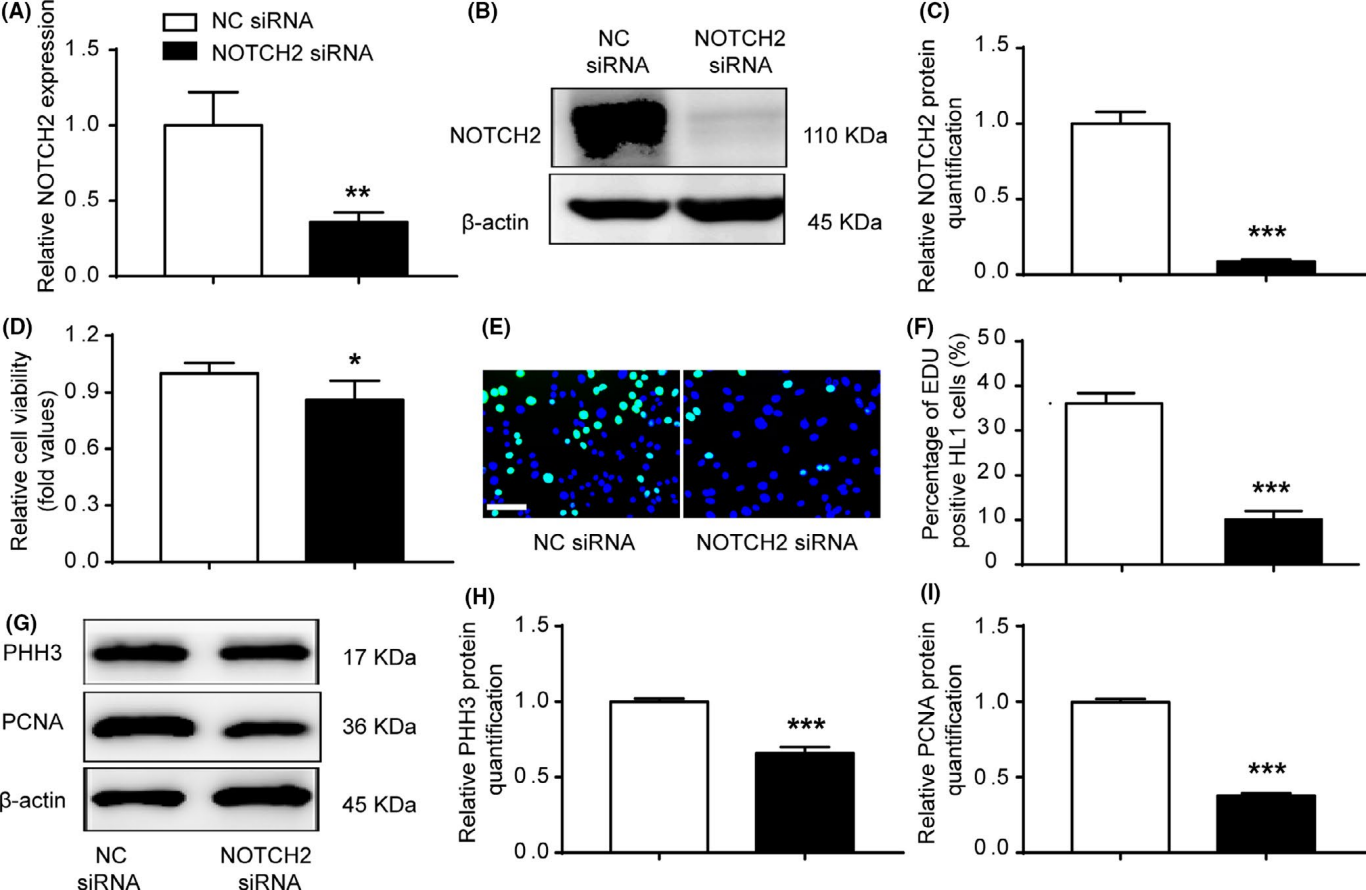
NOTCH2 siRNA inhibited cardiomyocyte proliferation. A‐C, qRT‐PCR and WB were performed in HL1 cells transfected with NOTCH2 siRNA. NOTCH2 siRNA decreased the endogenous NOTCH2 expression. D, Cell viability was examined via CCK‐8 assays. Cells transfected with NOTCH2 siRNA exhibited decreased cell viability compared with the control cells. E and F, EdU incorporation assay of cardiomyocytes. NOTCH2 siRNA decreased EdU incorporation (n > 1000, bar = 50 μm). G, WB analysis of PHH3 and PCNA. H and I, Relative quantification of PHH3 and PCNA protein. The expression of PHH3 and PCNA protein was down‐regulated in the NOTCH2 siRNA group. The data are presented as the mean ± SEM. Statistical significance is shown as *P < .05 vs controls, **P < .01 vs controls, and ***P < .001 vs controls

**Figure 7:**
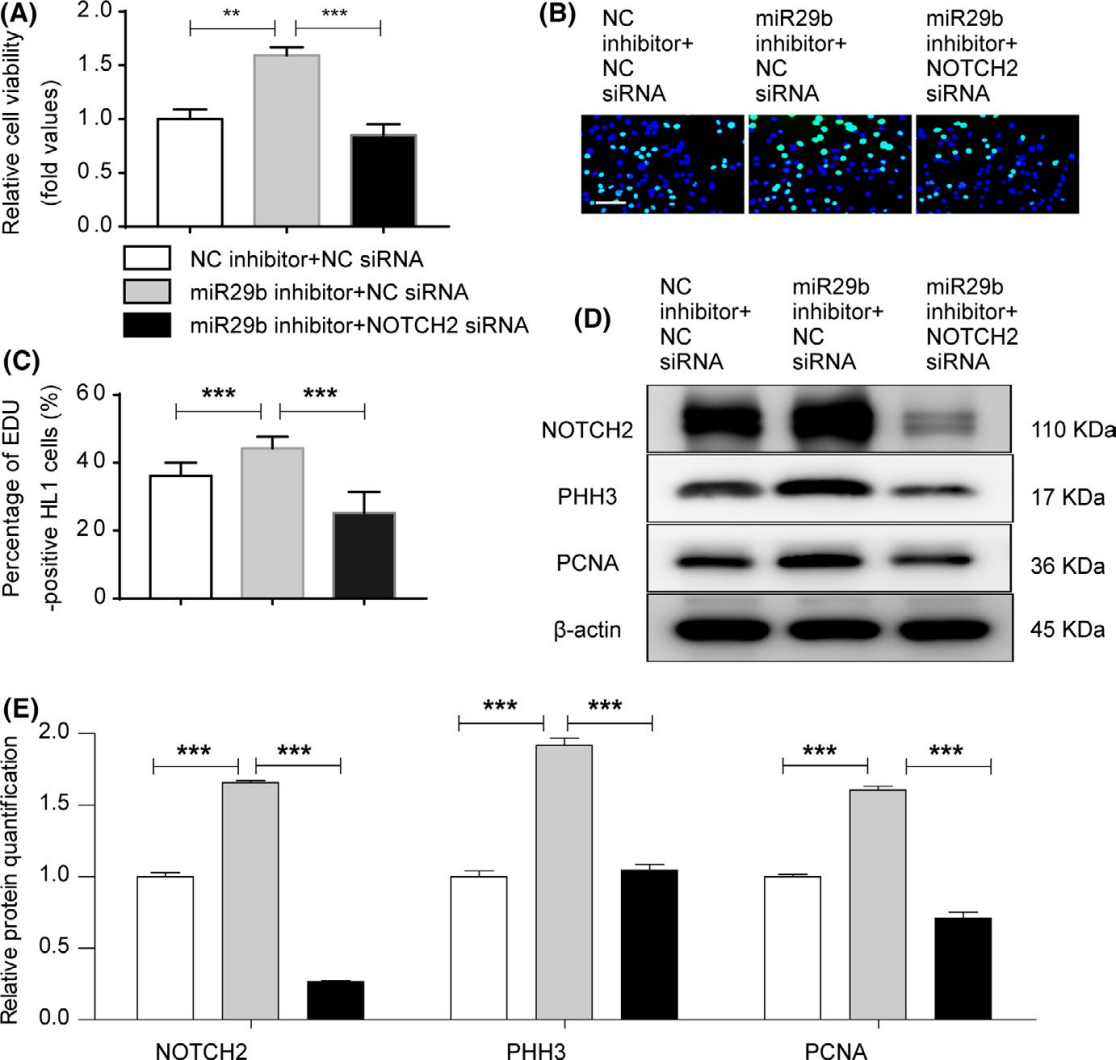
NOTCH2 siRNA partially weakened the pro‐proliferative effect of miR‐29b‐3p inhibitor. A, Cell viability was examined via CCK‐8 assays. Increased cell viability was observed in cells transfected with miR‐29b‐3p inhibitor, and co‐transfection with NOTCH2‐siRNA decreased cell viability. B and C, EdU incorporation assay of cardiomyocytes. Increased EdU incorporation was observed in the miR‐29b‐3p inhibitor group, and co‐transfection with NOTCH2‐siRNA decreased this change (n > 1000, bar = 50 μm). D, WB analysis of NOTCH2, PHH3 and PCNA. E, Relative quantification of NOTCH2, PHH3 and PCNA protein. The expression of NOTCH2, PHH3 and PCNA protein was higher in the miR‐29b‐3p inhibitor group and was decreased by co‐transfection with NOTCH2‐siRNA. The data are presented as the mean ± SEM. Statistical significance is shown as *P < .05 vs controls, **P < .01 vs controls, and ***P < .001 vs controls

To investigate whether miR‐29b‐3p mediated NOTCH2 repression could account for the anti‐proliferative effects of miR‐29b‐3p, we first detected the influence of NOTCH2 on cell proliferation. A significant decrease in NOTCH2 expression was observed in HL1 cells transfected with NOTCH2‐siRNA (Figure 6A‐C). NOTCH2‐siRNA significantly decreased cardiomyocyte viability and proliferation (Figure 6D‐F). NOTCH2 knockdown inhibited the expression of PHH3 and PCNA (Figure 6G‐I). These results suggested that NOTCH2 knockdown repressed cell proliferation. We co‐transfected cardiomyocytes with miR‐29b‐3p inhibitor and NOTCH2 siRNA and compared them with cells in the miR‐29b‐3p inhibitor group. Cardiomyocyte viability and DNA synthesis were lower in the co‐transfection group compared with the miR‐29b‐3p inhibitor group (Figure 7A‐C). In addition, NOTCH2 siRNA weakened the up‐regulation effect of miR‐29b‐3p inhibitor on the expression of NOTCH2, PHH3 and PCNA (Figure 7D,E).

**Figure 8:**
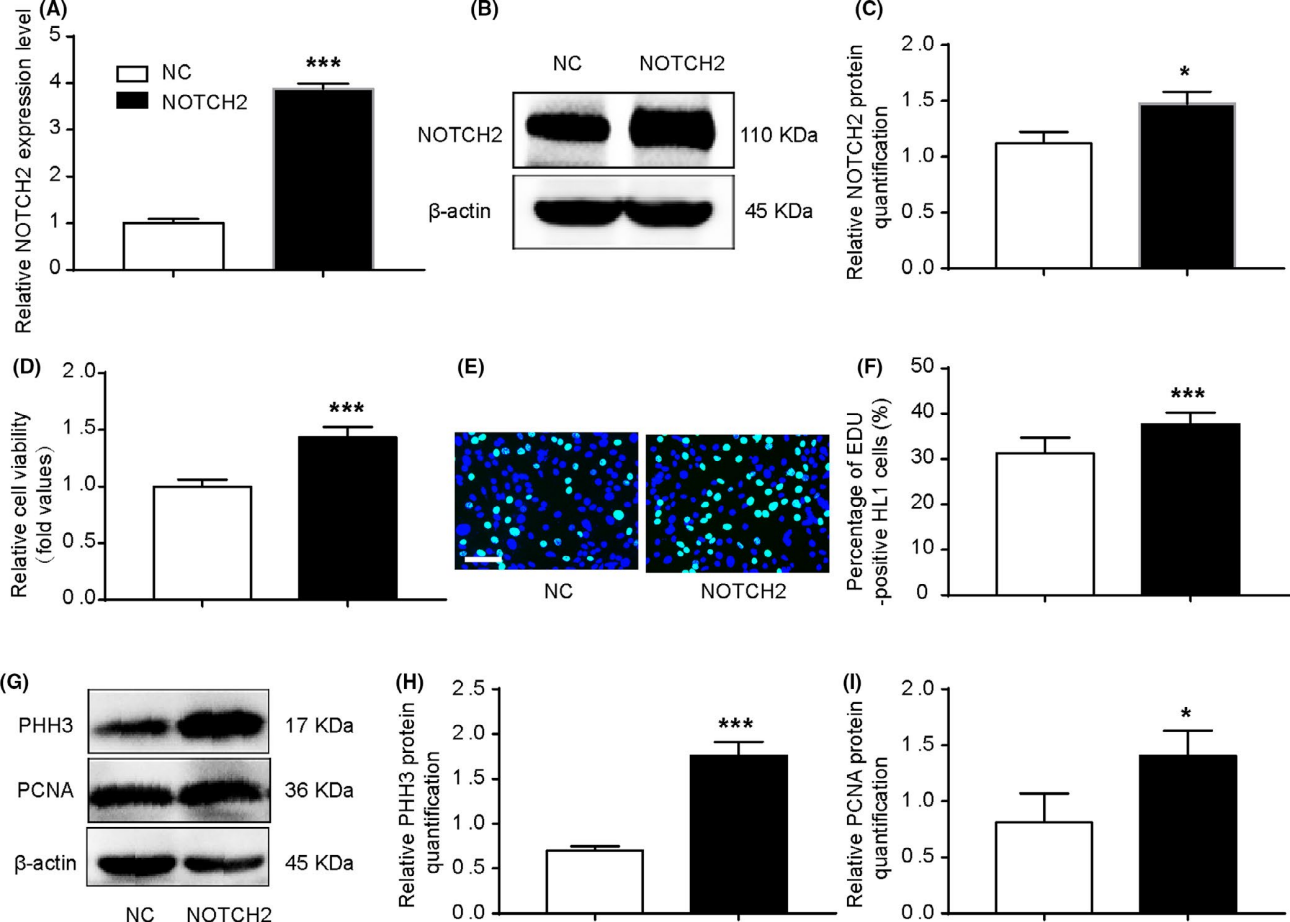
NOTCH2 promoted cardiomyocyte proliferation. A‐C, qRT‐PCR and WB were performed in HL1 cells transfected with pCHD‐NICD2‐puro plasmid. The plasmid increased the endogenous NOTCH2 expression. D, Cell viability was examined via CCK‐8 assays. Cells transfected with pCHD‐NICD2‐puro plasmid promoted cell viability compared with the control group. E and F, EdU incorporation assay of cardiomyocytes. Overexpression of NOTCH2 increased EdU incorporation (n > 1000, bar = 50 μm). G, WB analysis of PHH3 and PCNA. H and I, Relative quantification of PHH3 and PCNA protein. The expression of PHH3 and PCNA protein was up‐regulated in the NOTCH2 group. The data are presented as the mean ± SEM. Statistical significance is shown as *P < .05 vs controls, **P < .01 vs controls, and ***P < .001 vs controls

**Figure 9:**
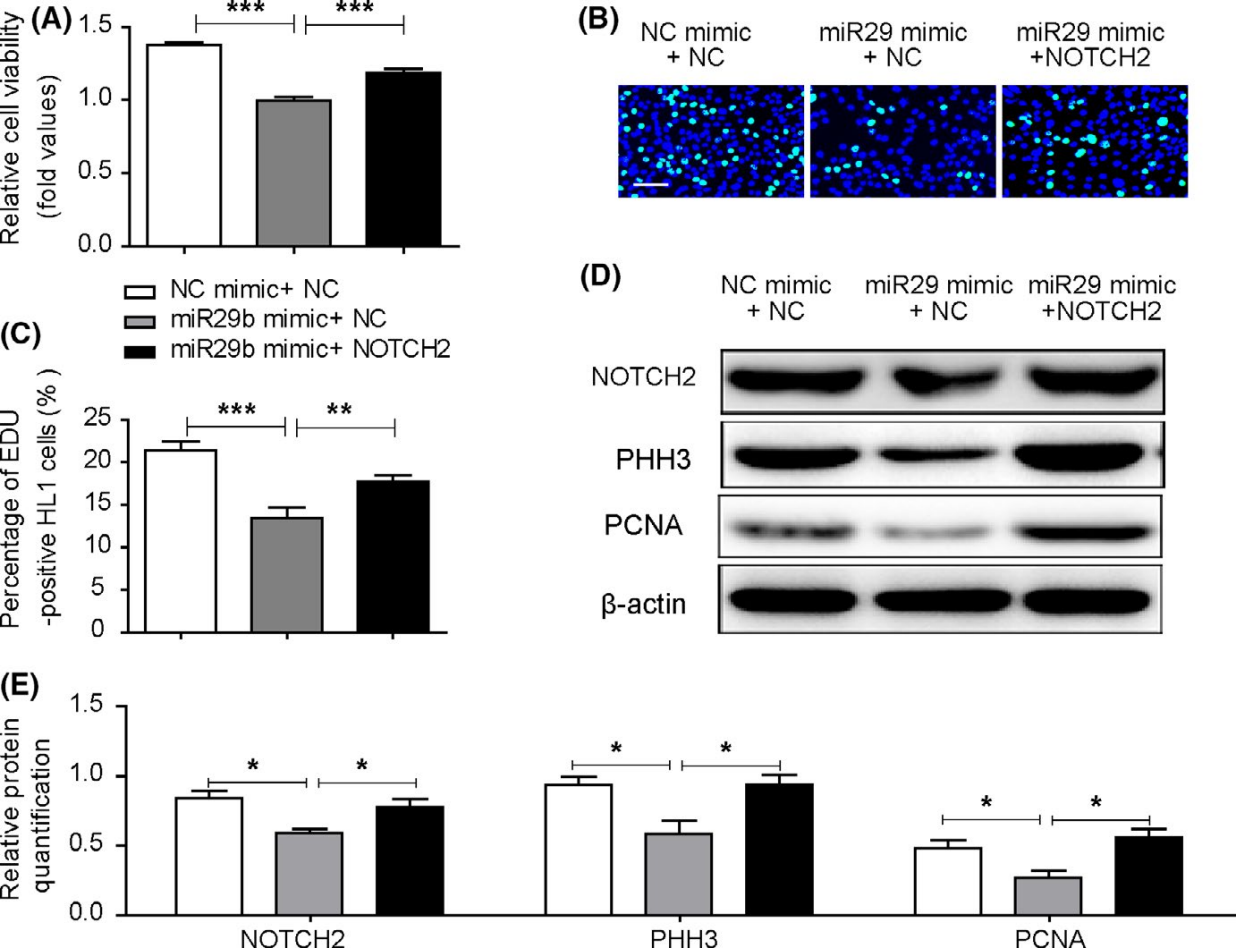
NOTCH2 attenuated the inhibitory effect of miR‐29b‐3p mimic on cell proliferation. A, Cell viability was examined via CCK‐8 assays. Decreased cell viability was observed in cells transfected with miR‐29b‐3p mimic, and co‐transfection with NOTCH2 increased cell viability. B and C, EdU incorporation assay of cardiomyocytes. Decreased EdU incorporation was observed in the miR‐29b‐3p mimic group, and co‐transfection with NOTCH2 decreased this change (n > 1000, bar = 50 μm). D, WB analysis of NOTCH2, PHH3 and PCNA. E, Relative quantification of NOTCH2, PHH3 and PCNA protein. The expression of NOTCH2, PHH3 and PCNA protein was lower in the miR‐29b‐3p mimic group and was increased by co‐transfection with NOTCH2. The data are presented as the mean ± SEM. Statistical significance is shown as *P < .05 vs controls, **P < .01 vs controls, and ***P < .001 vs controls

To further explore the role of NOTCH2 on cell proliferation, we transfected HL1 cells with pCHD‐NICD2‐puro plasmid, which showed significant increase in NOTCH2 expression (Figure 8A‐C). NOTCH2 promoted cardiomyocytes viability and DNA synthesis (Figure 8D‐F). Furthermore, NOTCH2 overexpression enhanced the expression of PHH3 and PCNA (Figure 8G‐I). These results suggested that NOTCH2 overexpression promoted cell proliferation. We co‐transfected cardiomyocytes with pCHD‐NICD2‐puro plasmid and miR‐29b‐3p mimic. It was found that NOTCH2 attenuated the inhibitory effect of miR‐29b‐3p mimic on cell proliferation (Figure 9A‐E). Overall, miR‐29b‐3p plays an important role on cell proliferation by targeting NOTCH2.

## 4 DISCUSSION

Numerous studies have suggested that miRNAs play a vital role in cardiac development, but only a few have described the miRNAs involved in its pathogenesis. In our study, we found that proper miR‐29b‐3p expression was important for cardiac development, and we explored the molecular mechanism by which miR‐29b‐3p regulated cardiomyocyte proliferation. First, we found that the expression of miR‐29b‐3p was significantly higher in the RVOT of CHD patients. Second, injection of miR‐29b‐3p induced cardiac malformation and dysfunction and inhibited cardiomyocyte proliferation in vivo and in vitro. Finally, we identified that the effect of miR‐29b‐3p on proliferation was partly mediated by NOTCH2, which was down‐regulated in the RVOT of the CHD patients. Hence, we conclude that miR‐29b‐3p regulates heart development and cardiomyocyte proliferation by targeting NOTCH2.

Several researchers have performed miRNA microarray analyses to compare the miRNA expression pattern in CHD patients with normal tissues,^17^, ^18^, ^19^ but there are just a few overlapping miRNAs among the results, which might due to different ages and races of patients, varying pre‐surgical and surgical treatments, or differences in the severity of the disease.^19^ Members of the miR‐29 family were differentially expressed in CHD patients, and their function may not be identical. However, none of these studies verified the expression of different mature forms of miR‐29 family via qRT‐PCR. In our study, we identified that miR‐29b‐3p was overexpressed in the RVOT tissue of CHD patients, which indicated its potential role in cardiac development.

MiR‐29b targets multiple genes, such as collagens, transcription factors and DNA methyltransferases, which participate in cell migration, invasion, apoptosis and proliferation. MiR‐29b was down‐regulated and recognized as tumour suppresser factors in many cancers, but it mediated epithelial‐mesenchymal transition and promoted metastasis in breast cancer and colon cancer.^21^ In our study, we found that miR‐29b‐3p was a potent inhibitor of cardiomyocyte proliferation in vitro and in vivo. These results suggest that the function of miR‐29b varies in different diseases and tissue types, which might be due to its specific target genes. Thus, identification of tissue‐specific targets to illustrate the pathogenic mechanism of miR‐29b in different diseases is important.

We found that miR‐29b‐3p directly targeted NOTCH2, a key regulator of heart development. NOTCH signalling is an evolutionarily conserved pathway and aberrations in NOTCH pathways lead to the occurrence of heart malformation,^22^ such as bicuspid aortic valve disease, calcification of the heart valves, Alagille syndrome and VSD.^23^ Researchers found that mutations in Jagged1 and NOTCH2 contributed to Alagille syndrome, an autosomal dominant genetic disorder that resulted in pulmonary artery stenosis and CHD.^24^, ^25^ Moreover, the Jag1^dDSL^/+ Notch2^del1^/+ double heterozygous mice exhibited right ventricular hypoplasia, pulmonary stenosis, OA, VSD and ASD.^26^ Using Cre‐lox technology to inhibit NOTCH signalling specifically in the neural crest cells (NCCs) resulted in outflow tract defects, including pulmonary stenosis, OA, VSD and ASD.^27^ Hes1 was a downstream mediator of the Notch signalling pathway and Hes1 mutant mice at embryonic day 15.5 exhibited outflow tract defects including VSD and OA. At earlier developmental stages (E9.5 and E10.5), Hes1 mutant mice embryos showed defects in the proliferation of SHF, a reduction in the number of NCCs and failure to completely extend the outflow tract.^28^ The down‐regulated expression of NOTCH2 in the CHD group may provide insight into the pathogenic mechanism of CHD.

As a member of the NOTCH receptor family, NOTCH2 plays an important role in a variety of physiological and pathological processes. Several studies have shown that NOTCH2 effectively modulates cell proliferation. Giachino et al revealed that NOTCH signalling cooperates with p53 to restrict cell proliferation and tumour growth in mice.^29^ Conversely, Dill et al reported that constitutive NOTCH2 signalling in mice was sufficient to induce hepatocellular carcinoma and biliary hyperplasia.^30^ Some researchers have reported that NOTCH2 silencing induced cell cycle arrest at G0/G1 phase in human glioma cells.^31^, ^32^ NOTCH2 not only functions in cancer but also regulates the vital events of cardiovascular development. The outflow tract is composed of migratory cardiac progenitor cells, including NCCs, SHF and resident primary heart field cells,^33^ and NOTCH signalling influences the proliferation and differentiation of cardiac progenitors. Altered NOTCH signalling in NCCs resulted in cardiac outflow tract malformations. NOTCH signalling promoted NCCs proliferation, and its inhibition resulted in decreased proliferation of NCCs.^34^ Moreover, Varadkar et al demonstrated that NOTCH2 was essential to the proliferation of cardiac neural crest‐derived smooth muscle cells for proper formation of the heart outflow tract.^35^ Ward et al reported that 76% of the chick embryos with ablated SHF at HH14 exhibited cardiac defects including pulmonary atresia and OA, while the embryos ablated at HH18 maintained normal outflow alignment. These results indicated that the myocardium added to the outflow tract by the proliferating SHF at earlier stages, which was required for the elongation and appropriate alignment of the outflow tract.^11^ Our results showed that miR‐29b‐3p influences cardiac development and regulates cardiomyocyte proliferation via targeting NOTCH2, which proved the important role of NOTCH2 on heart development.

The precursors of miR‐29b‐3p include miR‐29b‐1 and miR‐29b‐2. MiR‐29b‐1 are located on Chr. 7q32.3, while miR‐29b‐2 is located on Chr. 1q32.2. MiR‐29b not only plays an important role in cardiac fibrosis, aortic aneurysms and vascular integrity,^36^, ^37^ but also functions in cell proliferation regulation through complex signalling pathways. Wang et al identified a NF‐κB‐YY1‐miR‐29 regulatory circuit in rhabdomyosarcoma (RMS) pathogenesis. Activation of the NF‐κB‐YY1 pathway inhibited miR‐29 in RMS, and reconstitution of miR‐29 stimulated differentiation, suggesting that miR‐29 acted as a tumour suppressor through its pro‐myogenic function.^38^ Tumaneng et al reported that YAP mediates crosstalk between the Hippo and PI3K‐TOR pathways by suppressing PTEN via miR‐29, and PTEN was identified as a tumour suppressor gene.^39^ Another study found that miR‐29‐mediated Akt3 reduction in myoblasts could delay cell proliferation, while Akt3 overexpression inhibited myoblast differentiation.^40^ Our results suggest that miR‐29b‐3p inhibits cardiomyocyte proliferation by directly suppressing the NOTCH2. Overall, miR‐29b‐3p can regulate multiple pathways that might converge on a common biological outcome, such as proliferation regulation.

MiRNAs regulate multiple targets and bind to the same targets simultaneously, allowing for a complex pattern of regulation of gene expression.^41^ Many researches have explored the relationship between miRNAs and NOTCH2. Table 2 showed the miRNAs that have been validated to target NOTCH2.^42^, ^43^, ^44^, ^45^, ^46^, ^47^, ^48^, ^49^ MiRNAs inhibited the expression of NOTCH2 by binding their seed sequences with the 3′ UTR of the target genes. None of these miRNAs showed significant difference in expression between CHD patients and the controls.^18^, ^19^, ^50^ Among these miRNAs, miR‐1 is the most widely studied miRNA that involved in heart development and highly expressed in the RVOT. We validated the expression of miR‐1 in our heart tissues by qRT‐PCR and found no difference between the CHD group and the control group (Figure S7).

**Table 2:** The miRNAs that target NOTCH2

miRNA	Target	Cell type
miR‐1^42^	NOTCH2	Oesophageal squamous cell carcinoma cells
miR‐375^43^	NOTCH2	P19 cells
miR‐18a‐5p^44^	NOTCH2	Human aortic valvular endothelial cells
miR‐107^45^	NOTCH2	Glioma cells
miR‐34a^46^	NOTCH2	Glioma cells
miR‐205^47^	NOTCH2	Mammary epithelial cells
miR‐23b^48^	NOTCH2	Gastric cancer cells
miR‐146a; miR‐21^49^	NOTCH2	Vascular smooth muscle cells

Our results suggested that injection of miR‐29b‐3p induced abnormal heart morphology and function in zebrafish embryos. Although the zebrafish heart is a two‐chamber organ, it exhibits many similarities with the mammalian heart. Furthermore, cellular and molecular studies have illustrated the common evolutionary origin of these structures. Modern transcriptome analyses show that ~96% of genes associated with human cardiomyopathy are expressed in the zebrafish heart.^51^ A myriad of reporter lines exists to image not only structural and congenital heart disease, including ciliopathies, heterotaxy, RASopathies, cohesinopathies, mitral valve prolapse, endocardial cushion defects, septal defects, diGeorge syndrome and tetralogy of Fallot,^52^, ^53^, ^54^, ^55^, ^56^, ^57^ but also physiology such as electrical conduction, myocardial contraction, and more.^58^, ^59^, ^60^

These findings that aberrant expression of miR‐29b‐3p influences cardiac development and regulates cardiomyocyte proliferation via targeting NOTCH2 underscore the importance of epigenetic factors in the pathogenesis of CHD. To conclude, miR‐29b‐3p influences cardiac development and cardiomyocyte proliferation through repression of the NOTCH2, and these findings provide novel insight into the aetiology and potential treatments of CHD.

## CONFLICT OF INTEREST

None declared.

## AUTHOR CONTRIBUTIONS

Yonghao Gui and Qiang Li directed the experimental study. Qian Yang performed the experiments, analysed the data and wrote the manuscript. Fang Wu and Yaping Mi collected the heart tissues. Fang Wu, Yaping Mi, Feng Wang, Ke Cai and Xiaoshan Yang participated in the detection of cardiomyocyte proliferation in vitro. Ranran Zhang, Lian Liu and Yawen Zhang participated in the detection of cell proliferation in zebrafish. Youhua Wan provided technical support. Xu Wang and Mingqing Xu contributed to the language editing. Mingqing Xu participated in the revision of this article.

## Data Availability

The data that support the findings of this study are available from the corresponding author upon reasonable request.
