# Supplementary figures and images for: Aberrant expression of miR‐29b‐3p influences heart development and cardiomyocyte proliferation by targeting NOTCH2
Source: Cell Prolif. 2020 Feb 20;53(3):e12764. doi: 10.1111/cpr.12764 (PMC7106969; doi:10.1111/cpr.12764)

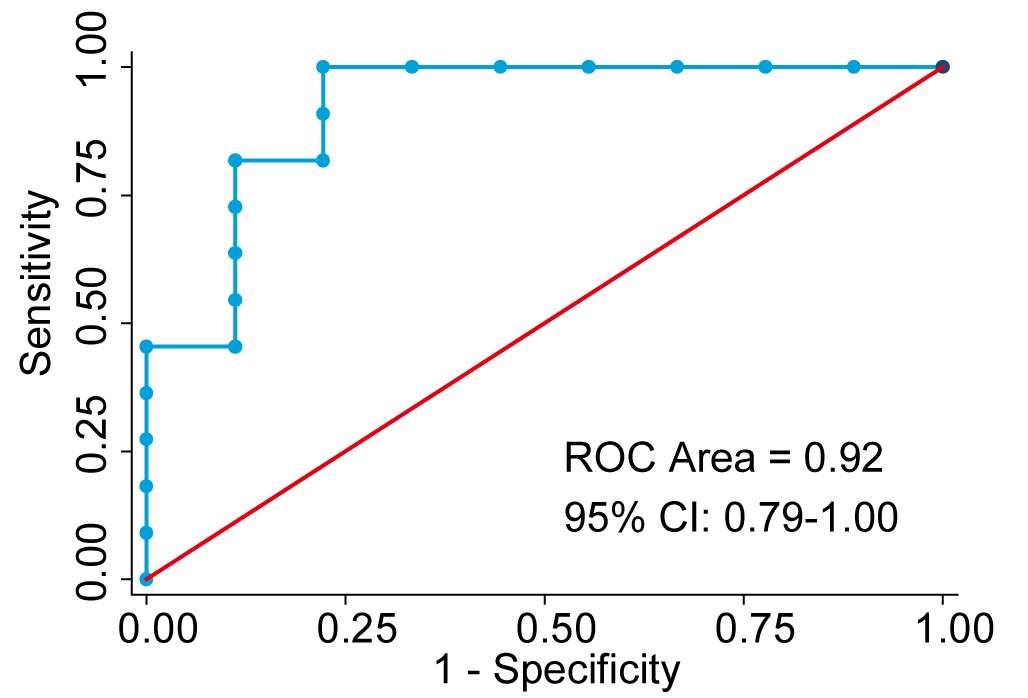

Supplement: Supplementary file 1 [file CPR-53-e12764-s001.tif]

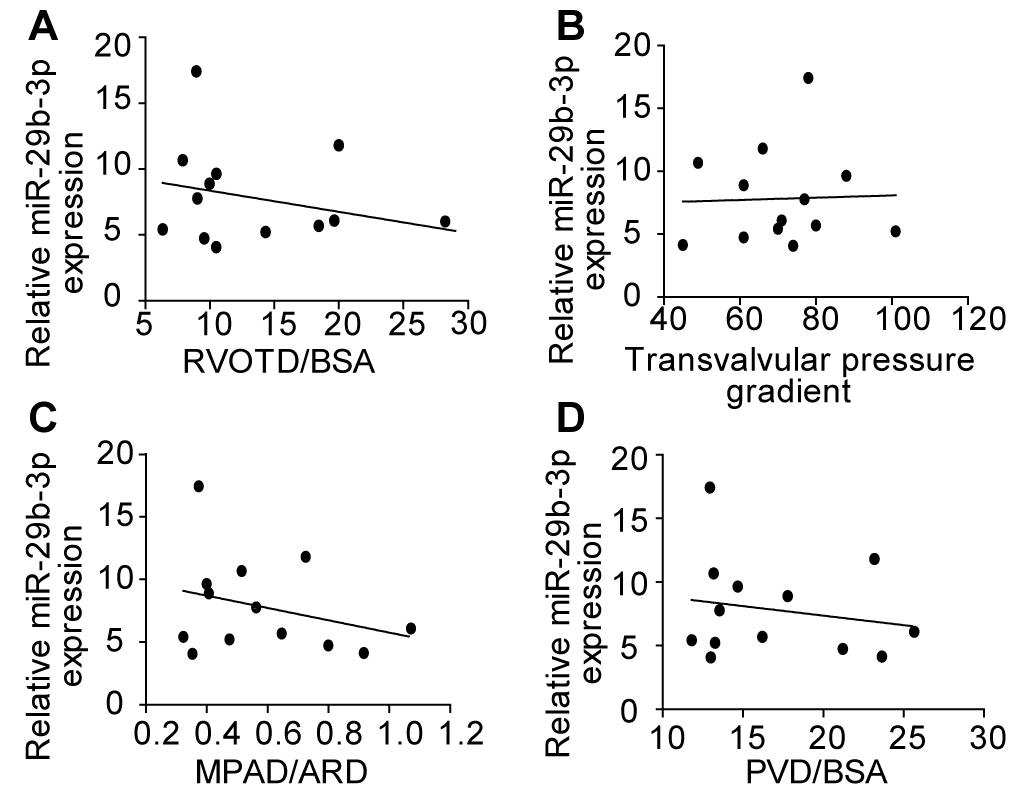

Supplement: Supplementary file 2 [file CPR-53-e12764-s002.tif]

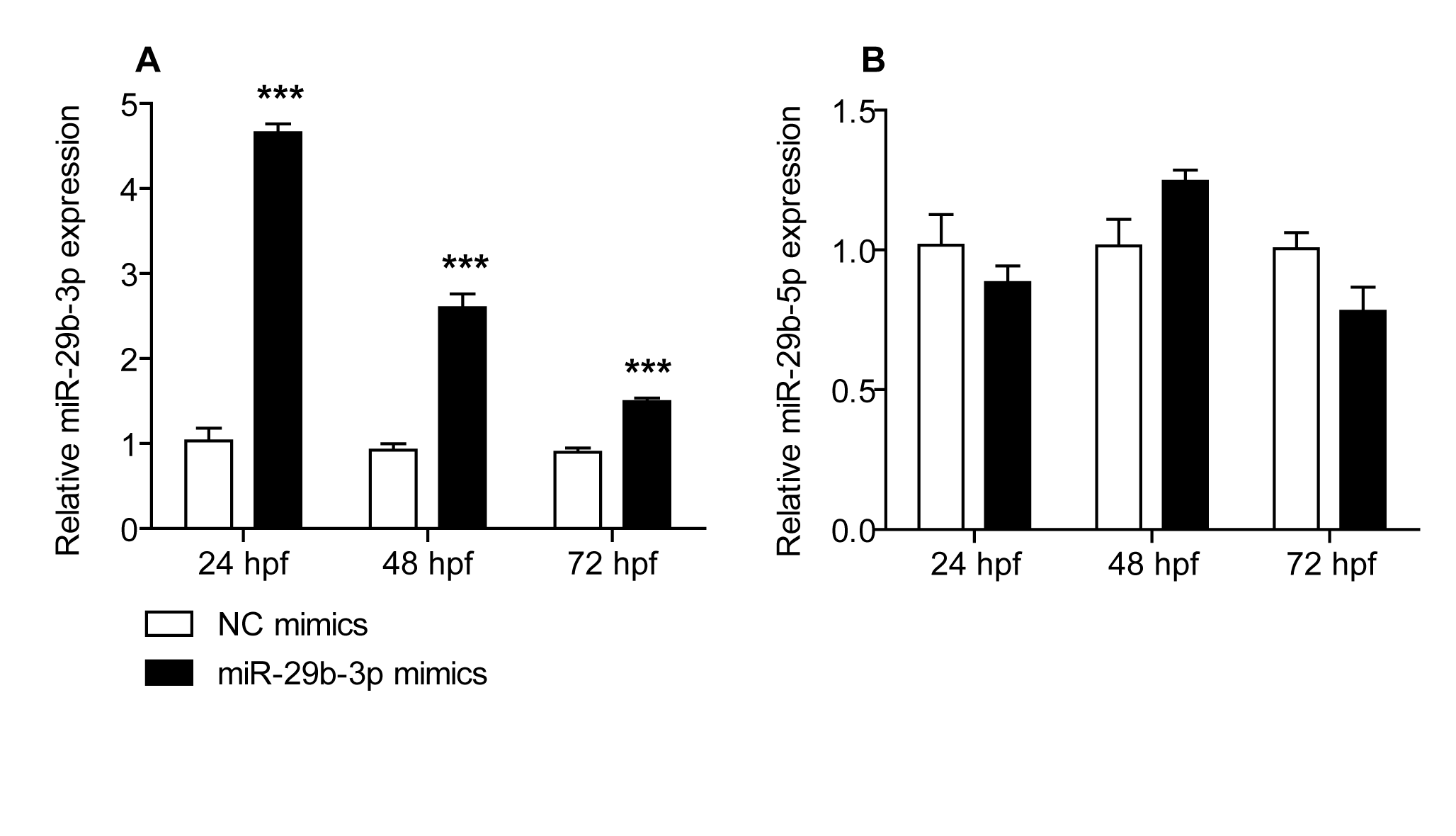

Supplement: Supplementary file 3 [file CPR-53-e12764-s003.tif]

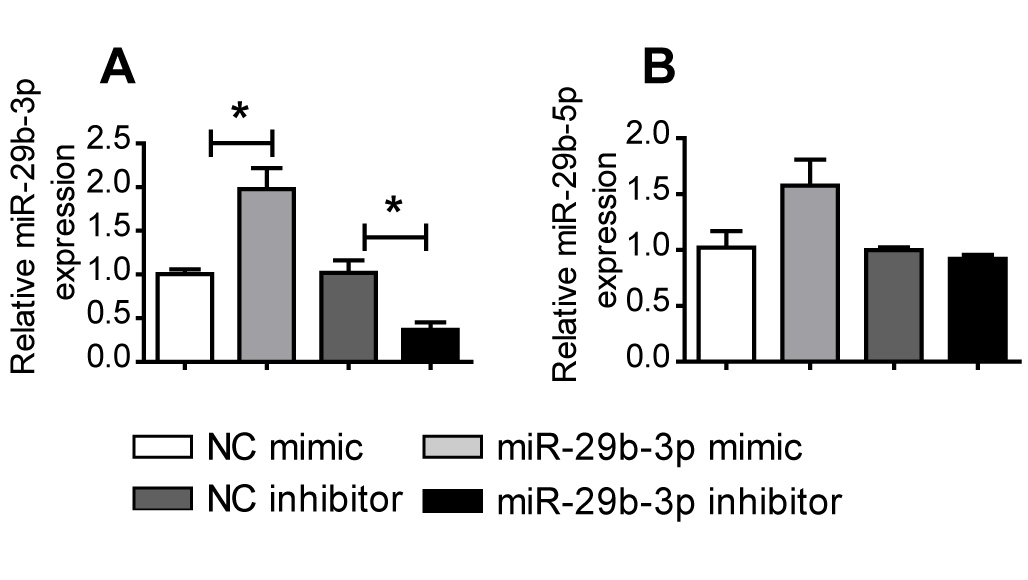

Supplement: Supplementary file 4 [file CPR-53-e12764-s004.tif]

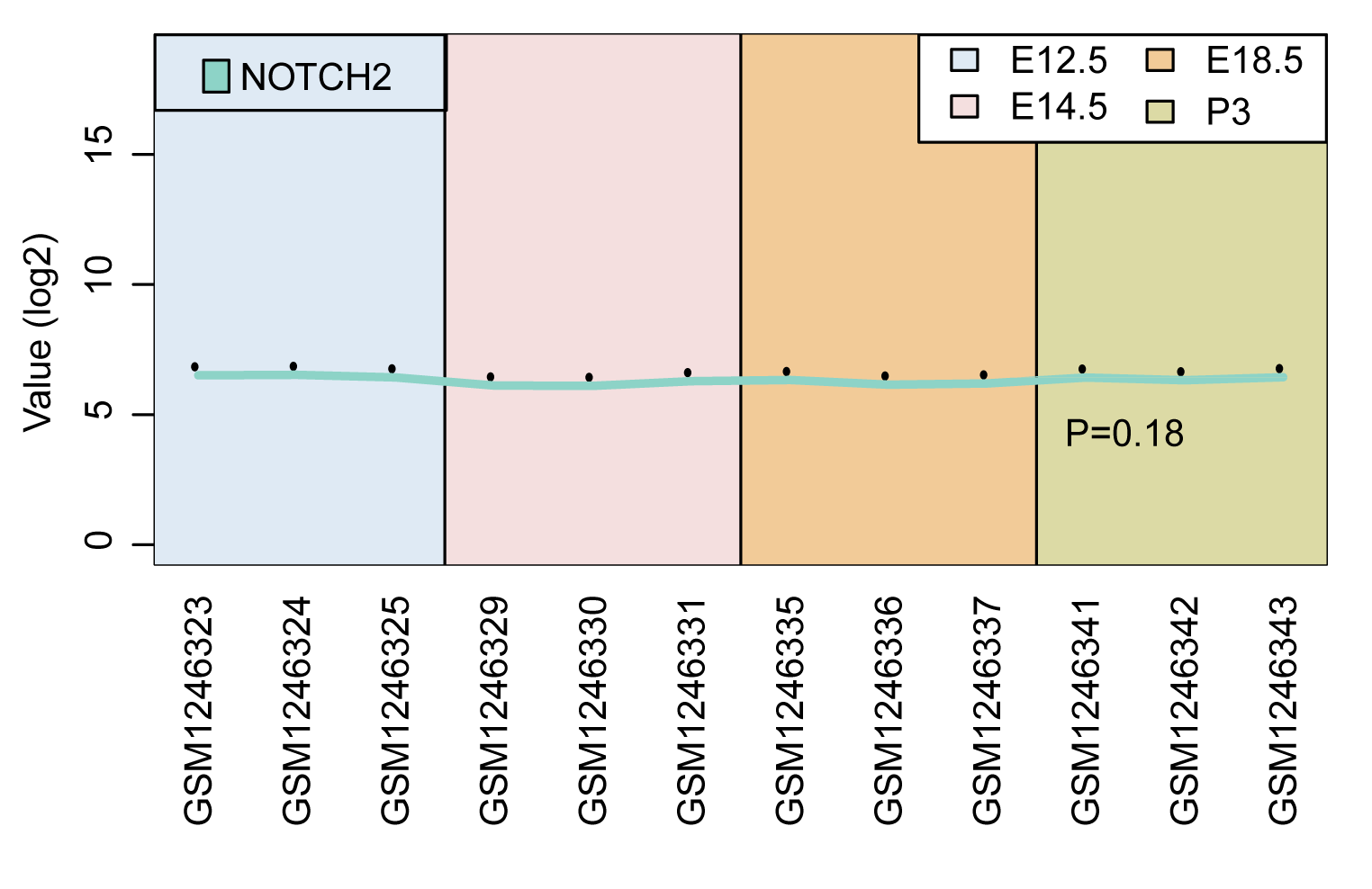

Supplement: Supplementary file 5 [file CPR-53-e12764-s005.tif]

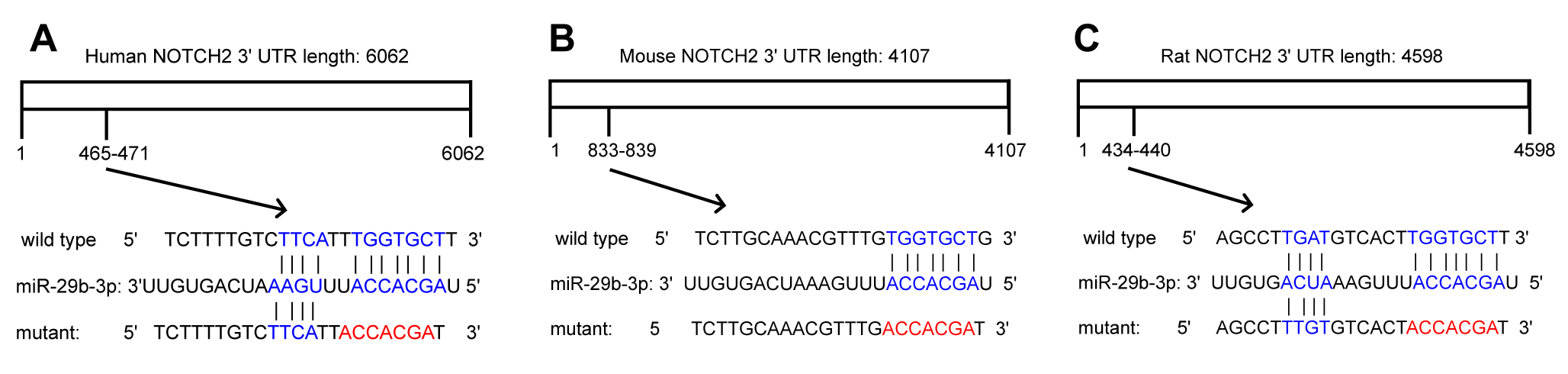

Supplement: Supplementary file 6 [file CPR-53-e12764-s006.tif]

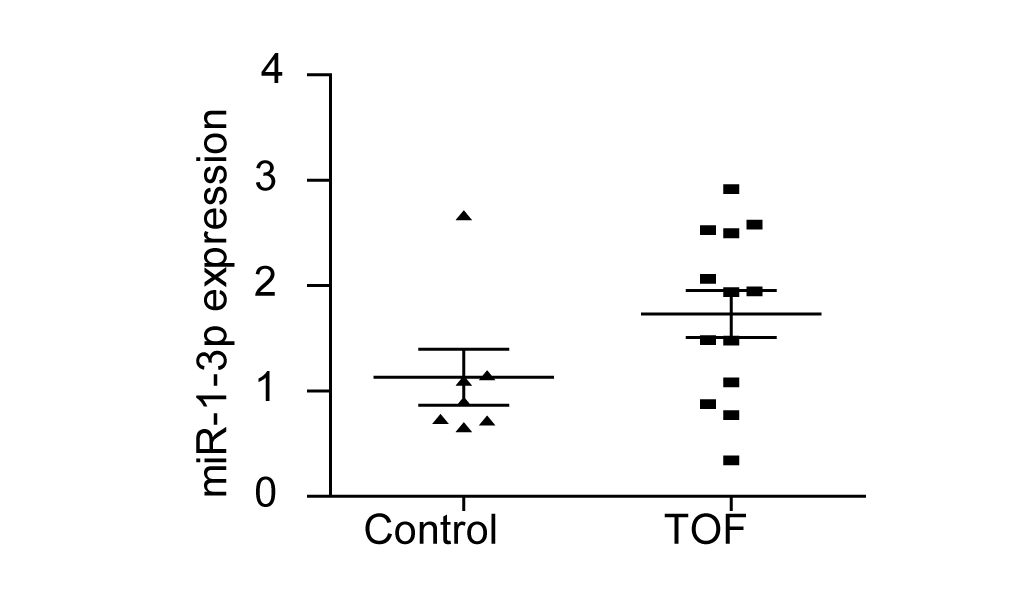

Supplement: Supplementary file 7 [file CPR-53-e12764-s007.tif]
