# Supplementary material for: Aberrant expression of miR‐29b‐3p influences heart development and cardiomyocyte proliferation by targeting NOTCH2
Source: Cell Prolif. 2020 Feb 20;53(3):e12764. doi: 10.1111/cpr.12764 (PMC7106969; doi:10.1111/cpr.12764)
Supplement: Supplementary file 8 [file CPR-53-e12764-s008.docx]

**Supplemental Table 1. Information of control group and CHD patients**

| **No** | **Age** | **Gender** | **Death cause** | **Clinical character** |
| --- | --- | --- | --- | --- |
| Control 1 | 23w | female | spontaneous abortion | --- |
| Control 2 | 25w | male | spontaneous abortion | --- |
| Control 3 | 23w | female | spontaneous abortion | --- |
| Control 4 | 27w | female | spontaneous abortion | --- |
| Control 5 | 27w | male | spontaneous abortion | --- |
| Control 6 | 26w | female | spontaneous abortion | --- |
| Control 7 | 26w | male | spontaneous abortion | --- |
| Case 1 | 2.75y | female | --- | TOF |
| Case 2 | 0.75y | male | --- | TOF+PFO |
| Case 3 | 0.33y | male | --- | TOF+PDA+PFO |
| Case 4 | 0.67y | female | --- | TOF+ASD |
| Case 5 | 4.83y | female | --- | TOF+PFO |
| Case 6 | 0.33y | female | --- | TOF+PDA+PFO |
| Case 7 | 0.58y | male | --- | TOF+PFO |
| Case 8 | 4y | male | --- | TOF |
| Case 9 | 1y | female | --- | TOF |
| Case 10 | 0.58y | male | --- | TOF+PFO |
| Case 11 | 1.58y | female | --- | TOF |
| Case 12 | 4.75y | male | --- | TOF |
| Case 13 | 1.25y | male | --- | TOF |

TOF: Tetralogy of Fallot

PFO: Patent foramen oval

PDA: Patent ductus arteriosus

ASD: Atrial septal defect

**Supplemental Table 2.** **Quantitative RT-PCR primers**

| **Primer name** | **Primer sequence** |
| --- | --- |
| COL6A2-Human | F: 5-AGCAGGAGGTCATCTCGCCG-3; |
|  | R: 5-GGATGTCCGTGGGGGACTGC-3; |
| NOTCH2-Human | F: 5-TCAACTGCCAAGCGGATGT-3; |
|  | R: 5-CTTGGCTGCTTCATAGCTCC-3; |
| NRAS-Human | F: 5-CCTAAATCTGTCCAAAGCAGAGGCAG-3; |
|  | R: 5--GGATTAGCTGGATTGTCAGTGCGC-3; |
| NTRK3-Human | F: 5-CCGCGATGGTTTCAGACGCTG-3; |
|  | R: 5-GCCCACATAGTCCAGCCAGACG-3; |
| SNIP1-Human | F: 5-TGCGGTCTTTCAATATCGGC-3; |
|  | R: 5-AGAAGGTTCCATTGCCTGAGC-3; |
| VEGFA-Human | F: 5-CAGCCCGAGCCGGAGAGG-3; |
|  | R: 5-CTGCCATGGGTGCAGCCTG-3; |
| β-actin-Human | F: 5-CTGACGGCCAGGTCATCAC-3; |
|  | R: 5-CAGACAGCACTGTGTTGGC-3; |
| NOTCH2-Mouse | F: 5-CCACCTGCCTGGATAAGATCG-3; |
|  | R: 5-CTGCCCGTTGTTCACACAC-3; |
| β-actin-Mouse | F: 5-ATATCGCTGCGCTGGTCGTC-3; |
|  | R: 5-AGGATGGCGTGAGGGAGAGC-3; |
| NOTCH2-Rat | F: 5-CAGTGTCGAGGTGGTCAAGAGCC-3; |
|  | R: 5-CACAAGTACCACCATTCTGACAGCG-3; |
| β-actin-Rat | F: 5-CACCCGCGAGTACAACCTTC-3; |
|  | R: 5-CCCATACCCACCATCACACC-3; |
| NOTCH2-Zebrafish | \| F: 5-GCCAATTACCCGGTGTCTCTTCG-3; \| \| --- \| |
|  | R: 5-GCAGTAGTGTGTCCCGTTGCTTGAG-3; |
| β-actin-Zebrafish | F: 5-TGGAGAAGAGCTACGAGCTCC-3; |
|  | R: 5-CCGCAGGACTCCATTCCGAG-3; |

**Supplemental Table 3.**   **The sequence of miR-29b-3p**

| miRNAs | Accession | Sequence |
| --- | --- | --- |
| hsa-miR-29b-3p | MIMAT0000100 | UAGCACCAUUUGAAAUCAGUGUU |
| rno-miR-29b-3p | MIMAT0000801 | UAGCACCAUUUGAAAUCAGUGUU |
| mmu-miR-29b-3p | MIMAT0000127 | UAGCACCAUUUGAAAUCAGUGUU |
| **dre-miR-29b3-3p** | **MIMAT0048668** | **UAGCACCAUUUGAAAUCAGUGUU** |

**Supplemental Table 4. The sequences of miRNA mimic and inhibitor**

| **miRNA name** | **Sequence** |
| --- | --- |
| NC mimic | Sense: 5-UUCUCCGAACGUGUCACGUTT-3; |
|  | antisense: 5-ACGUGACACGUUCGGAGAATT-3; |
| miR-29b-3p mimic | Sense: 5-UAGCACCAUUUGAAAUCAGUGUU-3; |
|  | antisense: 5-CACUGAUUUCAAAUGGUGCUAUU-3; |
| NC inhibitor | 5-CAGUACUUUUGUGUAGUACAA-3; |
| miR-29b-3p inhibitor | 5-AACACUGAUUUCAAAUGGUGCUA-3; |

**Supplemental Table 5.** **psiCHECK-2-NOTCH2-3’ UTR vector primers**

| **Primer name** | **Primer sequence** |
| --- | --- |
| NOTCH2-3’UTR -WT-Human | F: 5- AGAGCTCGAGCTCTGGAGCCAGCTTCTAGAGGTAGGA-3; |
|  | R: 5-AGAGGCGGCCGCCCGGTATCCCTTGGAGTCTCACAAG-3; |
| NOTCH2-3’UTR -MUT-Human | F: 5-GACATTCTTTTGTCTTCATT ACCACGATTTGGTTTTG-3; |
|  | R: 5-TCGTGGTAATGAAGACAAAAGAATGTCCAAGGAGGAGG-3; |
| NOTCH2-3’UTR -WT-Mouse | F: 5-AGAGCTCGAGGTGATAGCCTACTGTGCCCTTCCC-3; |
|  | R: 5-AGAGGCGGCCGCGAGGCACTAAAGGCAGTGATCAGCTC-3; |
| NOTCH2-3’UTR -MUT-Mouse | F: 5-TCTTGCAAACGTTTGACCACGAGAATCTTGCT-3;  R: 5-TCGTGGTCAAACGTTTGCAAGAAGCAACTCCA-3; |
| NOTCH2-3’UTR -WT-Rat | F: 5-AGAGCTCGAGTGAAGAGACCTCTAACCAGCCTCTGG-3; |
|  | R: 5-AGAGGCGGCCGCACAGTGGAGATCAATGTTGGGCCT-3; |
| NOTCH2-3’UTR -MUT-Rat | F: 5-AGCCTTGATGTCACT ACCACGA TTCCTCTCTG-3; |
|  | R: 5-TCGTGGTAGTGACATCAAGGCTACTTCCAGGG-3; |
